# Cervical Spinal Epidural Abscess From Haemophilus influenzae in an Adult: A Case Report

**DOI:** 10.7759/cureus.74946

**Published:** 2024-12-02

**Authors:** Luxi Qiao, April Choi, Julianna Jung

**Affiliations:** 1 Department of Emergency Medicine, Johns Hopkins University School of Medicine, Baltimore, USA

**Keywords:** cervical pain, haemophilus influenzae, infectious disease, neck pain, neurologic emergency, pyogenic spinal infection, spinal epidural abscess

## Abstract

Pyogenic spinal infections due to *Haemophilus influenzae* (*H. influenzae*)* *are rare. After a search of the literature, we deemed our case to be the first description of spinal epidural abscess (SEA) from *H. influenzae*. This is a 74-year-old female patient with a history of diabetes who presented to the emergency department with fever and persistent paracervical pain after being initially diagnosed with viral sinusitis two days prior. The examination was negative for neurologic deficits. She was admitted for possible meningitis versus intramuscular abscess due to cerebrospinal fluid pleocytosis and CT imaging findings. However, an MRI obtained during the patient’s admission revealed a multi-level cervical SEA, likely from an invasive *H. influenzae* infection, and the patient underwent evacuation surgery with a good outcome. Epidural abscesses are rarely present in isolation in the cervical region and can be mistaken for other more common infectious and non-infectious causes of neck pain on initial presentation and even as diagnostic work-up progresses. However, there is significant morbidity if diagnosis via advanced imaging and surgical treatment is delayed. We aim to present the first reported case of SEA from *H. influenzae* and increase awareness of this atypical presentation of a rare yet invasive disease.

## Introduction

Patients presenting with neck pain and fever evoke a broad differential, including infectious meningitis, deep space neck infections such as retropharyngeal abscess, and pyogenic spinal infections such as vertebral osteomyelitis and epidural abscess. Spinal epidural abscess (SEA) is an uncommon diagnosis, with a prevalence rate reported to vary between 0.18 and 1.96 per 10,000 hospital admissions [1]. The difficulty in diagnosing SEA can lead to delayed or suboptimal management, resulting in adverse outcomes such as permanent neurologic deficits, paraplegia, or death [2]. The most common causative organism for SEA is *Staphylococcus aureus*, reported in 47.9% of cases, and other pathogens such as *Streptococcus* (5.7%), *Brucellosis* (4.7%), *Escherichia coli* (3.3%), *Pseudomonas *(1.9%), *Klebsiella *(1.4%), and *Enterococcus, Proteus, *and* Mycobacterium tuberculosis* have also been observed [1]. However, there is currently no documented case of SEA from *Haemophilus influenzae* (*H. influenzae*) [3]. Here, we present the first reported case of SEA associated with *H. influenzae* bacteremia, which was diagnosed only after multiple presentations to healthcare settings due to the delayed onset of traditional symptoms and signs and overlap with other etiologies. Given the potential for high morbidity and mortality associated with delayed diagnosis and treatment, this report highlights the need for physicians to recognize SEA as an invasive presentation of *H. influenzae* infection.

## Case presentation

A 74-year-old female with a history of hypertension, non-insulin-dependent diabetes, hyperlipidemia, and degenerative disc disease of the cervical spine presented to the emergency department (ED) after six days of neck pain and headache. The patient’s symptoms began with malaise, frontal headache, and nasal congestion. She then developed generalized myalgias, nausea, and one episode of emesis, which prompted her to visit a local urgent care two days before the presentation, where she was found to have leukocytosis and was sent to an outside ED for evaluation. In the outside ED, she endorsed lateral neck pain that was more severe on the left side and denied any vision changes, photophobia, fever, or chills. Vital signs were normal, and examination was notable for diffuse tenderness across bilateral trapezius and paracervical soft tissue with normal neck range of motion. Her sensation and motor function were intact. Laboratory studies were notable for a mild leukocytosis (Table 1), and a non-contrast CT scan of the head showed bilateral mastoid effusions, scattered mucosal thickening in the ethmoid air cells, and opacification of the right sphenoid sinus. The patient was diagnosed with acute on chronic neck strain and concomitant viral sinusitis and was discharged with supportive care.

**Table 1:** Complete blood count results from the patient's visit to outside emergency department

	Result	Reference range and units
White blood cell count	11.22	4.50-11.00 K/cu mm
Red blood cell count	4.67	4.00-5.20 M/cu mm
Hemoglobin	14.5	12.0-15.0 g/dL
Hematocrit	42.8	36.0-46.0%
Platelet count	129	150-350 K/cu mm
Neutrophil %	90.5	40.0-70.0%
Lymphocyte %	6.4	24.0-44.0%
Monocyte %	2.4	2.0-11. 0%
Eosinophil %	0.0	1.0-4.0%
Basophil %	0.3	0.0-2.0%

The patient then presented to our ED two days later with worsening neck pain and paresthesias in the bilateral upper extremities. She still endorsed an occipital headache but now also noted intermittent subjective fevers and chills, pain with neck movement, and decreased neck range of motion. She denied other symptoms.

Initial vital signs showed a temperature of 37°C (98.6°F) tympanic, a heart rate of 90, a blood pressure of 130/71, a respiratory rate of 18, and an oxygen saturation of 97% on room air. The patient was in no acute distress, and the neck was held in flexion toward the right with a decreased range of motion but no palpable swelling, warmth, or skin changes. There was tenderness to palpation of the left paracervical soft tissue and trapezius without midline cervical spine tenderness. Strength and sensation to light touch were intact and symmetrical in the bilateral upper and lower extremities.

Initial laboratory studies are presented below (Tables 2-3). Blood cultures were obtained. A repeat non-contrast CT scan of the head was unchanged from the prior. Given the patient’s severe lumbar spine scoliosis, interventional radiology was consulted to perform a fluoroscopy-guided lumbar puncture to rule out meningitis. Antibiotics were held pending cerebrospinal fluid (CSF) studies given stable vitals, a reassuring examination, and only mild leukocytosis. Fluids and additional analgesics were also given.

**Table 2:** Complete blood count results from the patient's visit to our emergency department

	Result	Reference range and units
White blood cell count	12.12	4.50-11.00 K/cu mm
Red blood cell count	4.42	4.00-5.20 M/cu mm
Hemoglobin	13.7	12.0-15.0 g/dL
Hematocrit	39.7	36.0-46.0%
Platelet count	92	150-350 K/cu mm
Neutrophil %	90	40.0-70.0%
Band %	1.0	2.0-6.0%
Lymphocyte %	4.0	24.0-44.0%
Monocyte %	2.0	2.0-11.9%
Eosinophil %	0.0	1.0-4.0%
Basophil %	0.0	0.0-2.0%

**Table 3:** Chemistry profile results from the patient's visit to our emergency department

	Result	Reference range and units
Sodium	130	135-148 mmol/L
Potassium	3.5	3.5-5.1 mmol/L
Chloride	93	96-109 mmol/L
Carbon dioxide	22	21-31 mmol/L
Urea nitrogen	23	7-22 mmol/L
Creatinine	0.8	0.5-1.2 mg/dL
Glucose	251	71-99 mg/dL
Calcium	8.9	8.4-10.5 mg/dL
Albumin	3.7	3.5-5.3 g/dL
C-reactive protein	32.8	≤0.5 mg/dL

After returning from lumbar puncture, the patient developed a muffled voice without drooling, trismus, respiratory distress, or odynophagia. The patient also developed a fever of 38.7°C (101.7°F) and tachycardia to the 110-130s, but the repeat neurologic examination was otherwise unchanged. A lactate obtained at this time was 4.5 (reference range and units: 0.5-2.0 mmol/L). Results from the CSF analysis are presented below (Table 4).

**Table 4:** CSF chemistry, cell-count, and microbiology analysis results VDRL: venereal disease research laboratory, HSV: herpes simplex virus, NAT: nucleic acid test, CSF: cerebrospinal fluid

	Result	Reference range and/or units
Appearance	Clear	-
Color	No color	-
White blood count, CSF	23	0-5 /cu mm
Red blood count, CSF	554	/cu mm
Neutrophil %, CSF	80	%
Lymphocyte %, CSF	11	%
Monocytes %, CSF	7	%
Eosinophils %, CSF	2	%
Polymorph absolute number, CSF	19	<4 /cu mm
Glucose, CSF	123	50-75 mg/dL
Protein, CSF	92.5	15.0-45.0 mg/dL
Gram stain and culture, CSF	Moderate polymorphonuclear leukocytes, no organisms seen	-
*Treponema pallidum* VDRL antibody screen, CSF	Antibodies not present	-
HSV-1 quantitative NAT, CSF	No DNA detected	-
HSV-2 quantitative NAT, CSF	No DNA detected	-

**Figure 1:**
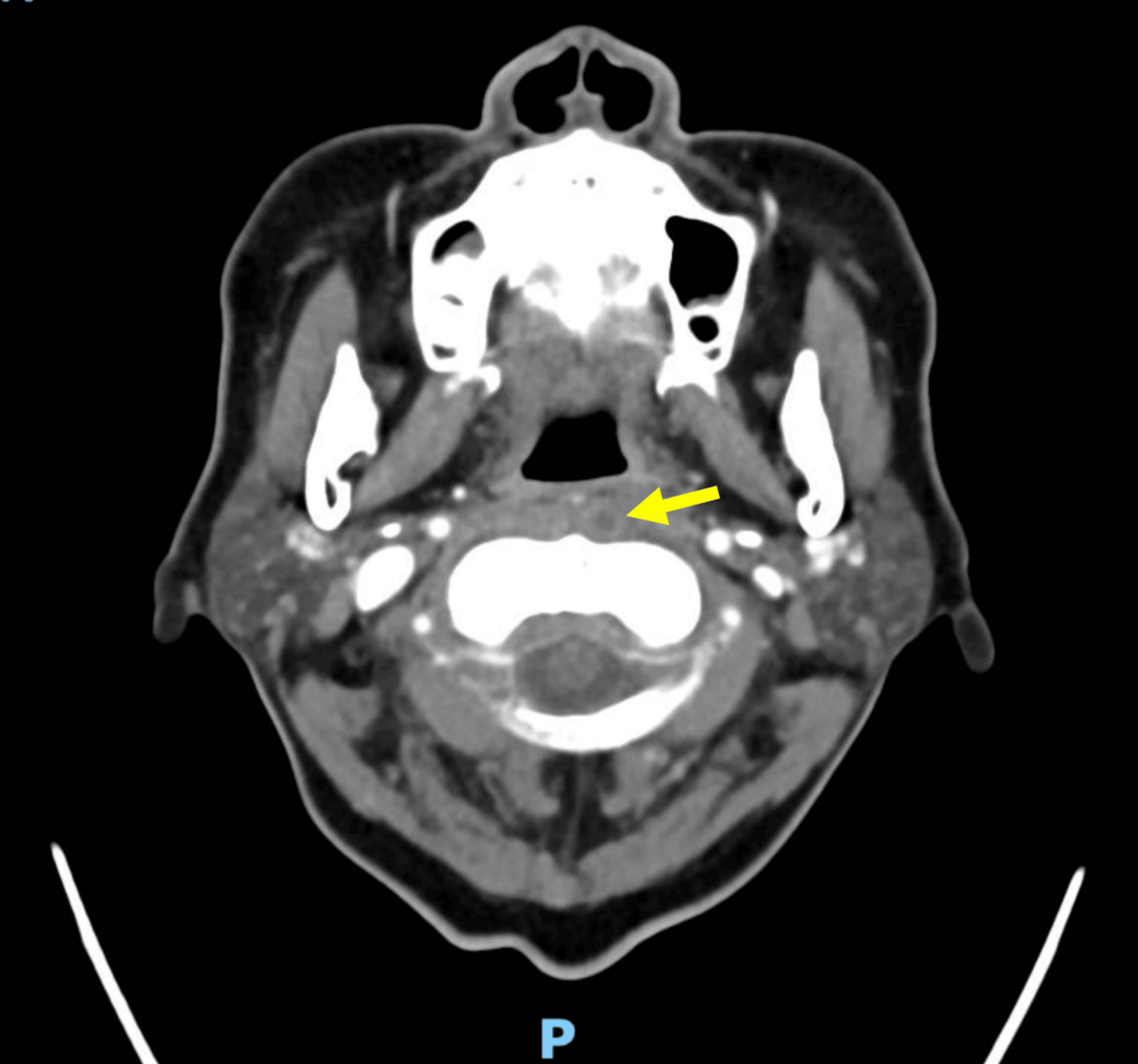
CT with intravenous contrast image of soft tissue of the neck (axial view) Rim-enhancing lesion measuring 8 mm in the left longus musculature at the C1-C2 level with associated retropharyngeal edema (yellow arrow). CT: computed tomography

A swab of the throat was negative for Group A *Streptococcus*. In the setting of the vital sign changes and CSF pleocytosis, there was a concern for meningitis as an infectious source. The patient was therefore started on broad-spectrum antibiotics consisting of ceftriaxone, ampicillin, and vancomycin. Due to the additional concern for retropharyngeal abscess, a CT scan of the neck with contrast was also obtained, which showed a ring-enhancing lesion in the left longus colli musculature at the C1-C2 level with associated retropharyngeal edema, possibly suggestive of calcific tendinitis of the longus muscle (Figure 1). The patient’s vital signs normalized shortly after initiating antibiotics, antipyretics, and additional fluid resuscitation, and the patient was admitted to the hospital.

**Figure 2:**
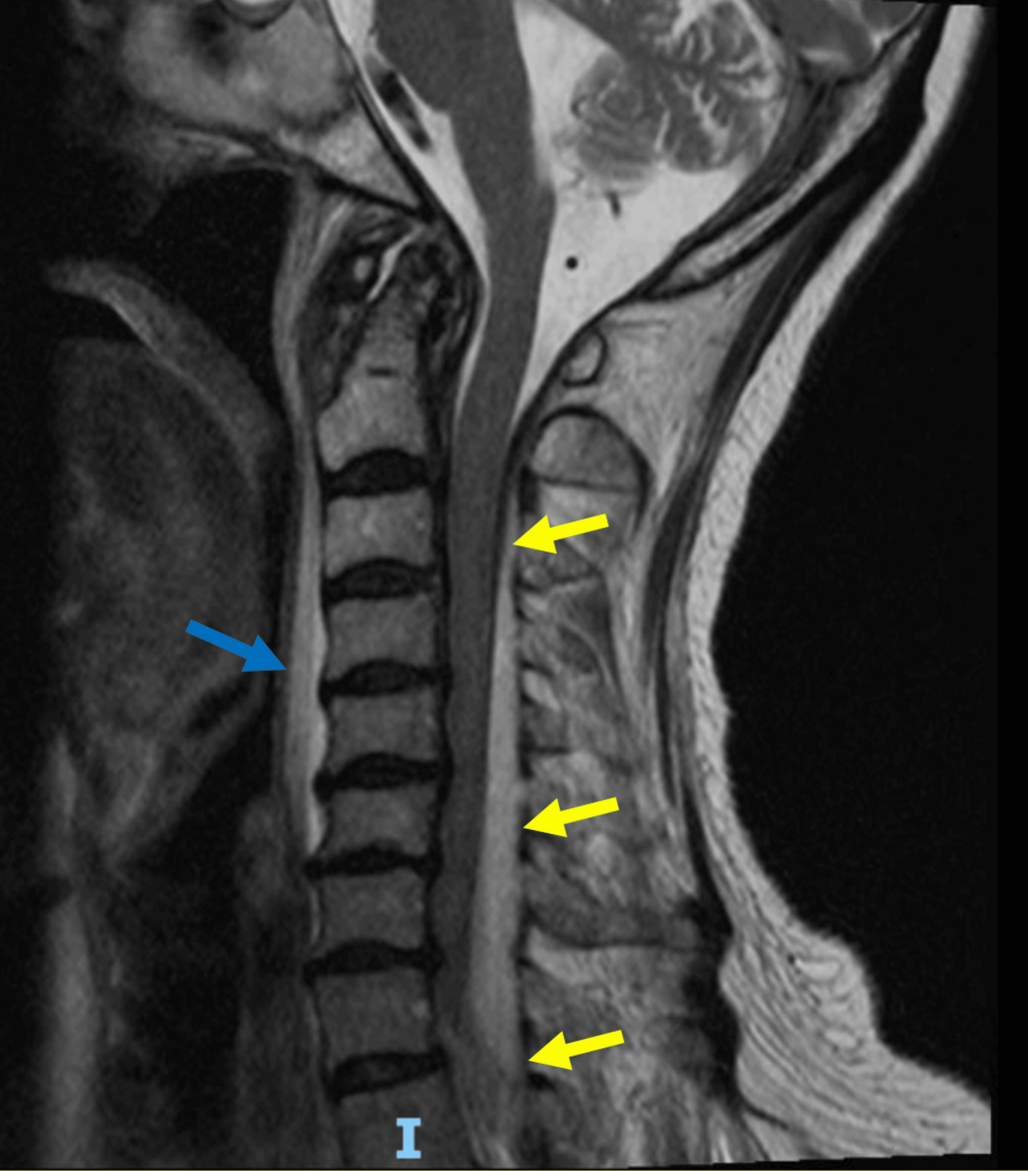
MRI T2-weighted image of the cervical spine (sagittal view) Rim-enhancing collection involving the dorsal epidural space, spanning from C2 level to inferior T1 level with mass effect on the thecal sac and cord most severely within the lower cervical spine (yellow arrow). Rim-enhancing lesion centered within the left longus coli musculature and associated extensive edema of the longus coli muscle and within the retropharyngeal space (blue arrow). MRI: magnetic resonance imaging

On hospital day 2, the patient’s blood cultures grew *H. influenzae*. The transthoracic echocardiogram did not show any valvular vegetations. Given the persistence of the neck pain and intermittent left upper extremity paresthesia, an MRI study was obtained to characterize the intramuscular lesion further. The study detected a dorsal SEA measuring 0.6 cm in thickness and spanning the entire cervical spine, causing spinal stenosis with cord compression that was most severe at the C7-T1 level (Figure 2). There was also an abscess within the left longus coli muscle measuring 0.8 cm transversely and 3.4 cm craniocaudally, as well as the retropharyngeal edema previously seen on CT. Orthopedic spine surgery evaluated the patient and performed urgent cord decompression and abscess drainage, with intraoperative findings of extensive purulence in the epidural space. The patient was also seen by interventional radiology and otorhinolaryngology, who could not identify any drainable fluid collection in the retropharyngeal space. The patient was then continued on ceftriaxone for six weeks. Intraoperative cultures were negative, though they were collected two days after initiating intravenous antibiotics. A repeat CT scan showed resolution of the intramuscular lesion, and the most recent outpatient follow-up with orthopedic spine surgery six months post-op noted she has no residual pain or neurologic deficits.

## Discussion

This case is atypical for several reasons. Firstly, the presentation is subtle. Our patient initially presented with neck pain and upper respiratory tract symptoms, and her neck pain was thought to be secondary to a viral syndrome given the associated sinusitis and lack of fever or significant leukocytosis. However, prior case series that examined patients with SEA in various sections of the spine have demonstrated that approximately 40% of the patients had normal WBC counts, and only 32% had a fever >38.4°C [4]. Rigamonti et al. also note that 29% of patients with cervical SEA had no motor deficits [4]. Thus, the first learning point from this case is that clinical findings such as fever, leukocytosis, and neurologic deficit are variably present in patients with cervical epidural abscess. On the second ED presentation, our patient endorsed intermittent bilateral upper extremity paresthesias, but this was thought to be secondary to her known degenerative disk disease. The rest of her neurologic examination was benign, and she did not have any symptoms of myelopathy, such as motor weakness, hyperreflexia, or bowel or bladder incontinence, which led us away from considering a diagnosis of SEA. A second learning point from our case is that patients with cervical SEA may have signs and symptoms that overlap with other causes of neck pain and fever. When our patient developed fever and tachycardia and CSF studies showed pleocytosis, an expanded work-up and treatment were appropriately initiated. Broad-spectrum intravenous antibiotics were administered to cover infectious meningitis, and the patient underwent more advanced imaging with a contrast CT study of the neck. CT noted retropharyngeal edema and potentially calcific tendinitis or abscess of the longus colli muscle. Vilke and Honingford reported a case of cervical epidural abscess that showed a fluid collection in the retropharyngeal space on CT. That patient was initially diagnosed with a retropharyngeal abscess until the development of motor deficits prompted myelography [5]. As in our case, the retropharyngeal findings were later attributed to an inflammatory response secondary to the SEA. In addition, our patient, like prior case reports, was evaluated and treated for meningitis due to CSF pleocytosis even though, ultimately, the CSF did not grow any organisms [5-7].

Another unique feature of this case is the causative organism, given the site of infection. *H. influenzae *is a non-motile, gram-negative, catalase-positive coccobacillus that primarily colonizes the human upper respiratory tract [8]. It is classified based on the presence or absence of serologically unique capsule polysaccharides, including six capsule serotypes (a to f) and nonencapsulated (non-typeable) forms [9]. Historically, *H. influenzae *serotype b (Hib) has been the primary cause of invasive infections, especially in the pediatric population. Since the introduction of the Hib vaccine in the 1980s, the epidemiology of *H. influenzae* infections has shifted to a higher proportion of non-b serotype and non-typable strains, affecting especially adults age 65 or older and children under one year of age [8]. Infection can present as sinusitis, otitis media, conjunctivitis, or invasive diseases such as pneumonia, bacteremia, meningitis, epiglottitis, osteomyelitis, and pericarditis [8]. An epidural abscess from *H. influenzae* has never been described, and even other types of pyogenic spinal infections due to *H. influenzae*, such as vertebral osteomyelitis or discitis, are exceedingly rare, with only 10 cases reported in the literature [3,10]. Most cases of SEA are considered to be secondary to hematogenous seeding, as presumably with our patient given the *H. influenzae* bacteremia, although direct extension has also been reported [11]. The most commonly reported underlying conditions for *H. influenzae* infection are chronic obstructive pulmonary disease, atherosclerotic cardiovascular disease, diabetes, and chronic heart failure [12]. Having diabetes likely also increased our patient’s susceptibility to SEA, as it is frequently cited as an associated risk factor. Existing literature also cites intravenous drug use, renal disease, and prior spinal procedures as other common risk factors for SEA [1].

SEA remains a rare diagnosis, though its incidence is on the rise, partly driven by the aging population [13,14]. This same demographic is also at heightened risk for *H. influenzae* infections [12]. SEA occurs less frequently in the cervical spine than in the lumbar or thoracic spine, ranging from 18% to 36% of cases [1]. However, it is consistently associated with worse neurological outcomes, and early surgical intervention, ideally before the onset of neurologic deficits, is crucial for improving patient outcomes [14]. While other etiologies of neck pain and fever, such as retropharyngeal abscess, meningitis, and epiglottitis, can all be confirmed via CT or lumbar puncture, detection of SEA requires an MRI. Management of SEA typically requires surgical debridement in addition to an extended course of intravenous antibiotics. It is particularly critical to keep this differential in mind in patients with relevant risk factors, neurologic complaints, or persistence of symptoms despite medical management.

## Conclusions

We present the first reported case of SEA associated with *H. influenzae* infection. Cervical SEA is often a difficult diagnosis as presenting symptoms can be variable, and many clinical, laboratory, and imaging features overlap with both benign and serious causes of neck pain and fever. MRI is required for diagnosis, and surgical management is typically needed in addition to parenteral antibiotics. Thus, when evaluating patients presenting with neck pain and fever or leukocytosis, physicians should keep this diagnosis in mind throughout the work-up and have a low threshold to obtain an MRI and surgical consultation. Our case not only contributes to the body of literature as the first reported case of SEA from *H. influenzae* but also serves to raise awareness about this uncommon yet deadly condition.
